# Lysosomal storage disorders for the pediatric rheumatologist: the example of mucopolysaccharydoses

**DOI:** 10.1186/1824-7288-41-S2-A17

**Published:** 2015-09-30

**Authors:** Rolando Cimaz, Angela Mauro

**Affiliations:** 1 Department of Pediatrics, Rheumatology Unit, AOU Meyer Hospital; Viale Pieraccini, n°24; 50139, Firenze, Italy

## 

The Mucopolysaccharidoses (MPS) are a group of diseases caused by complete or partial deficiency of lysosomal enzymes responsible for glycosaminoglycans catabolism. Their accumulation within the lysosome leads to cellular damage and organ failure [1,2].

The musculoskeletal system is the most frequently affected one. Joint stiffness, contractures (claw hand), dysostosis multiplex, and carpal tunnel syndrome are some of the most frequent features [3-9]. Often the joint symptoms may be confused with inflammatory arthritides such as Juvenile Idiopathic Arthritis [10,11]. A prompt differential diagnoses is a fundamental step: in MPS patients there are no signs of local inflammation such as swelling, warmth and tenderness, and lack of fever and increased inflammatory markers. In addition, patients with MPS do not respond to anti-rheumatic therapy [12,13].

In addition, characteristic facies, cognitive impairment, short stature, recurrent otitis media, sleep apnea, hearing, vision and heart problems can be present [14,15]. Because of a wide variety of clinical presentation, diagnosis of MPS disorders is often delayed, especially in patients with mild forms and without neurocognitive impairment such as Scheie Syndrome.

When clinical features are suggestive for MPS, the diagnosis is confirmed with the assay of urinary GAG concentration, which is a sensitive but not specific method [16] and, as the gold standard with determination of the specific enzyme in cultured fibroblasts, leukocytes, plasma or serum [17]. The genetic sequencing could be used to identify the disease-causing mutation.

The managment of MPS disorders requires a multidisciplinary evaluation for multi-organ involvement. The new therapeutic approaches to MPS have drastically changed the natural history of disease. Transplantation of hematopoietic stem cells from bone marrow or umbilical cord can achieve significant benefits. The clinical success of this therapeutic approach depends on age, stage of disease, type of donor and ability to achieve stable engraftment without the development of graft-vs-host disease [18,20]. In early stages of disease, enzyme replacement therapy can benefit musculoskeletal symptoms and lung function [21-23]. This therapy, however, does not cross the blood-brain barrier and has not shown neurocognitive benefit.

In conclusion, the goal is the prompt identification of MPS disorders since an early diagnosis could allow early treatment: in this regard, particular attention should be given to an accurate differential diagnosis with chronic inflammatory arthropathies.

## References

[B1] Muenzer J Overview of the mucopolysaccharidoses Rheumatology (Oxford) 2011 50 4 12 10.1093/rheumatology/keq312 22210669

[B2] Neufeld EU Muenzer J Scriver CR The mucopolysaccharidoses The Metabolic and Molecular Bases of Inherited Disease 2001 New York: McGraw-Hill 3421 52

[B3] Aldenhoven M Sakkers RJ Boelens J de Koning TJ Wulffraat NM Musculoskeletal manifestations of lysosomal storage disorders Ann Rheum Dis 2009 68 1659 65 10.1136/ard.2008.095315 19822711

[B4] Schmidt H Ullrich K von Lengerke HJ Kleine M Brämswig J Radiological findings in patients with mucopolysaccharidosis I H/S (Hurler-Scheie syndrome) Pediatr Radiol 1987 17 409 14 10.1007/BF02396619 3114705

[B5] Chen SJ Li YW Wang TR Hsu JC Bony changes in common mucopolysaccharidoses Zhonghua Min Guo Xiao Er Ke Yi Xue Hui Za Zhi 1996 37 178 84 8755171

[B6] Montano AM Tomatsu S Gottesman GS Smith M Orii T International Morquio A Registry: clinical manifestations and natural course of Morquio A disease J Inherit Metab Dis 2007 30 165 74 10.1007/s10545-007-0529-7 17347914

[B7] Mikles M Stanton RP A review of Morquio syndrome Am J Orthop 1997 26 533 40 9267552

[B8] Al-Qatan MM Thomson HG Clarke HM Carpal tunnel syndrome in children and adolescents with no history of trauma J Hand Surg 1996 21B 108 11 8676014 10.1016/s0266-7681(96)80023-4

[B9] Yuen A Dowling G Johnstone B Kornberg A Coombs C Carpal tunnel syndrome in children with mucopolysaccaridoses J Child Neurol 2007 22 260 3 10.1177/0883073807300528 17621494

[B10] Morishita K Petty RE Musculoskeletal manifestations of mucopolysaccharidoses Rheumatology (Oxford) 2011 50 19 25 10.1093/rheumatology/ker397 22210666

[B11] Petty RE Southwood TR Manners P Baum J Glass DN Goldenberg J International League of Associations for Rheumatology classification of juvenile idiopathic arthritis. second revision Edmonton, 2001 J Rheumatol 2004 31 390 2 14760812

[B12] Cimaz R Coppa GV Kone-Paut I Link B Pastores GM Elorduy MR Joint contractures in the absence of inflammation may indicate mucopolysaccharidosis Pediatr Rheumatol Online J 2009 7 18 10.1186/1546-0096-7-18 19852785 PMC2775028

[B13] Aldenhoven M Sakkers RJ Boelens J de Koning TJ Wulffraat NM Musculoskeletal manifestations of lysosomal storage disorders Ann Rheum Dis 2009 68 1659 65 10.1136/ard.2008.095315 19822711

[B14] Vijay S Wraith JE Clinical presentation and follow-up of patients with the attenuated phenotype of mucopolysaccharidosis type I Acta Paediatr 2005 94 872 7 10.1080/08035250510031584 16188808

[B15] Thomas JA Beck M Clarke JT Cox GF Childhood onset of Scheie syndrome, the attenuated form of mucopolysaccharidosis. I J Inherit Metab Dis 2010 33 421 7 20532982 10.1007/s10545-010-9113-7 PMC2903709

[B16] Mahalingam K Janani S Priya S Elango EM Sundari RM Diagnosis of mucopolysaccharidoses: how to avoid false positives and false negatives Indian J Pediatr 2004 71 29 32 10.1007/BF02725652 14979382

[B17] Hall CW Liebaers I Di Natale P Neufeld EF Enzymatic diagnosis of the genetic mucopolysaccharide storage disorders Methods Enzymol 1978 50 439 56 26836 10.1016/0076-6879(78)50048-7

[B18] Weisstein JS Delgado E Steinbach LS Hart K Packman S Musculoskeletal manifestations of Hurler syndrome: long-term follow-up after bone marrow transplantation J Pediatr Orthop 2004 24 97 101 10.1097/01241398-200401000-00019 14676543

[B19] Prasad VK Kurtzberg J Transplant outcomes in mucopolysaccharidoses Semin Hematol 2010 47 59 69 10.1053/j.seminhematol.2009.10.008 20109613

[B20] Boelens JJ Wynn RF O'Meara A Veys P Bertrand Y Souillet G Outcomes of hematopoietic stem cell transplantation for Hurler's syndrome in Europe: a risk factor analysis for graft failure Bone Marrow Transplant 2007 40 225 33 10.1038/sj.bmt.1705718 17529997 PMC7094454

[B21] McGill JJ Inwood AC Coman DJ Lipke ML de Lore D Swiedler SJ Enzyme replacement therapy for mucopolysaccharidosis VI from 8 weeks of age-a sibling control study Clin Genet 2010 77 492 8 10.1111/j.1399-0004.2009.01324.x 19968667

[B22] Schulze-Frenking G Jones SA Roberts J Beck M Wraith JE Effects of enzyme replacement therapy on growth in patients with mucopolysaccharidosis type II J Inherit Metab Dis 2011 34 203 8 10.1007/s10545-010-9215-2 20978944 PMC3026660

[B23] Wraith JE Beck M Lane R van der Ploeg A Shapiro E Xue Y Enzyme replacement therapy in patients who have mucopolysaccharidosis I and are younger than 5 years: results of a multinational study of recombinant human alpha-L-iduronidase (laronidase) Pediatrics 2007 120 e37 46 10.1542/peds.2006-2156 17606547

